# Per-fraction positional and dosimetric performance of prone breast tangential radiotherapy on Halcyon™ linear accelerator assessed with daily rapid kilo-voltage cone beam computed tomography: a single-institution pilot study

**DOI:** 10.1186/s13014-020-01700-6

**Published:** 2020-11-07

**Authors:** Suk W. Yoon, Neil K. Taunk, Gary M. Freedman, Emily Hubley, Shannon O’Reilly, Boon- Keng K. Teo, Shibu Anamalayil, Lei Dong, Christopher Kennedy, Wei Zou, James M. Metz, Taoran Li

**Affiliations:** Department of Radiation Oncology, Hospital of the University of Pennsylvania, Perelman Center for Advanced Medicine, 3400 Civic Center Blvd, Philadelphia, PA USA

**Keywords:** Whole breast radiation therapy, Tangential field radiotherapy, Adaptive replanning, O-ring linear accelerator, Daily CBCT

## Abstract

**Background:**

This study investigates daily breast geometry and delivered dose to prone-positioned patients undergoing tangential whole breast radiation therapy (WBRT) on an O-ring linear accelerator with 6X flattening filter free mode (6X-FFF), planned with electronic compensation (ECOMP) method. Most practices rely on skin marks or daily planar image matching for prone breast WBRT. This system provides low dose daily CBCT, which was used to study daily robustness of delivered dose parameters for prone-positioned WBRT.

**Methods:**

Eight patients treated with 16-fraction prone-breast WBRT were retrospectively studied. Planning CTs were deformed to daily CBCT to generate daily synthetic CTs, on which delivered dose distributions were calculated. A total of 8 × 16 = 128 synthetic CTs were generated. Consensus ASTRO definition was used to contour Breast PTV Eval for each daily deformed CT. Breast PTV Eval coverage (V90%) and hotspot (V105% and Dmax) were monitored daily to compare prescription dose with daily delivered dose. Various predictors including patient weight, breast width diameter (BWD), and Dice similarity coefficient (DSC) were fit into an analysis of covariance model predicting V90% and V105% deviation from prescribed (ΔV90%, ΔV105%). Statistical significance is indicated with asterisks (* for *p* < 0.05; ** for *p* < 0.001).

**Results:**

Daily delivered Breast PTV Eval V90% was moderately smaller than prescribed (median ΔV90% = − 0.1%*), while V105% was much larger (median ΔV105% = + 10.1%** or + 92.4 cc**). Patient’s weight loss correlated with significantly increased ΔV105% (+ 4.6%/ − 1% weight, R^2^ = 0.4**) and moderately decreased ΔV90% (− 0.071%/ − 1% wt., R^2^ = 0.2**). Comprehensive ANCOVA models indicated three factors affect ΔV90% and ΔV105% the most: (1) BWD decrease (− 0.09%* and + 10%**/ − 1 cm respectively), (2) PTV Eval volume decrease (− 0.4%** and + 9%**/ − 100 cc), and for ΔV105% only, (3) the extent of breast deformation (+ 10%**/ − 0.01 DSC). Breast PTV Eval volume also decreased with time (− 2.21*cc/fx), possibly indicating seroma resolution and increase in V105% over time.

**Conclusions:**

Daily CBCT revealed key delivered dose parameters vary significantly for patients undergoing tangential prone breast WBRT planned with ECOMP using 6X-FFF. Patient weight, BWD, and breast shape deformation could be used to predict dosimetric variations from prescribed. Preliminary findings suggest an adaptive plan based on daily CBCT could reduce excessive dose to the breast.

## Introduction

Breast cancer is diagnosed in about a quarter of a million women in the United States every year [1]. Lumpectomy with tangential-field, whole-breast radiotherapy (WBRT) is a standard treatment as part of breast conservation therapy (BCT) for early stage disease [2, 3]. Tangent WBRT can be delivered in either supine or prone position. Prone position may be optimal for patients with larger and more pendulous breasts for three reasons: (1) Reduced dose to heart and lungs [4], (2) respiratory motion of the chest walls and surgically inserted clip is reduced, consequently decreasing intra-fraction dosimetric variations [5], and (3) dose inhomogeneities in supine position raises the risk of late skin effects resulting in adverse cosmesis.

Daily image guidance is crucial in prone patient positioning, since immobilization devices alone cannot provide consistent day-to-day breast localization. Matching clips or bony/soft tissue anatomy with orthogonal electronic portal image devices (EPID) is a common standard for image guidance in prone WBRT. EPID positional accuracy is comparable to kV cone-beam CT (CBCT) guidance [6, 7]. Unlike EPID, however, CBCT can provide tomographic image from which 3-dimensional positional and dosimetric outcomes can be derived. Despite this advantage, busy clinics are discouraged from adopting daily CBCT because CBCT acquisition time may decrease throughput and increase imaging dose.

Halcyon™ v2.0 (Varian Medical Systems, Palo Alto, CA) is a commercially available 6MV flattening-filter-free O-ring linear accelerator that provides rapid daily kV CBCT image guidance for tangential WBRT. The O-ring design reduces the risk of collisions and enables higher gantry rotation speed for faster CBCT imaging, which demonstratively improved patient throughput in many clinical sites including whole breast [8, 9]. Compared to C-arm linacs, Halcyon v2.0 CBCT acquisition is faster (17–42 s versus 60 s), even after taking into account reconstruction duration for iterative CBCT, or iCBCT [10], which results in a better contrast-to-noise ratio.

One trade-off for higher patient throughput with Halcyon is the restriction to 6X-FFF beams, which results in a non-flat dose profile at depth. Electronic tissue compensation (ECOMP) planning technique [11] is a forward-planning technique that compensates for this non-flat profile to homogenously treat an irregular surface (i.e. breast) using parallel opposed beams. In contrast, Field-in-field (FiF) is a more common planning technique for C-arm linacs. It has been previously demonstrated that FiF takes significantly longer on Halcyon (9 min FiF vs. 3–4 min ECOMP) owing to Halcyon’s dual multi-leaf collimator (MLC) system.

It is unknown if ECOMP with FFF beam for prone WBRT meets dose coverage metrics (i.e. V90%) or hotspot metrics (i.e. V105%) on a per-fraction basis. American Society for Radiation Oncology (ASTRO) guidelines on whole breast RT state that V105% (volume of breast receiving 105% of prescription dose) should be minimized at all times [12], as V105% has been linked to adverse cosmesis [13]. ASTRO guidelines also state that V95% should cover the whole breast fully. The rapid CBCT capabilities of Halcyon v2.0 enables large-scale studies of dosimetric robustness, for example, by deforming planning CT to daily CBCT. Such capabilities can guide adaptive radiotherapy decisions if needed should plans fail to meet the guidelines.

In this paper, we assess the daily positional and dosimetric quality of ECOMP with FFF beam for tangential-beam prone WBRT, using per-fraction CBCTs provided by Halcyon v2.0 during patient positioning to: (1) Assess daily dosimetric and positional robustness of our treatments using daily CBCTs in real patient cases, and (2) investigate what factors affect dose homogeneity most adversely. Though the scope of this paper is limited to a single institution with limited number of patients, assessment of these factors may possibly inform future adaptive RT decisions and provide foundation for larger studies comparing Halcyon treatments with other treatment techniques.

## Methods

### Overall design of the study

The purpose of this study is to evaluate daily positional and dosimetric accuracy of tangential-field prone WBRT, planned with ECOMP and treated with 6X-FFF beam on Halcyon™ version 2.0 (Varian Medical Systems, Palo Alto, CA). We seek to investigate what parameters affect target coverage and radiation hotspot the most, using Halcyon’s rapid daily CBCT capabilities. This retrospective study was reviewed and approved by our Institutional Review Board.

Image acquisition was done as follows. Eight (8) prone breast patients receiving WBRT post-lumpectomy with hypofractionated schedule of 2.66 Gy × 16 fractions followed by 10 Gy boost were selected sequentially in the 6-month period from a pool of treated patients under IRB approval. There were no exclusion criteria other than ensuring that none of the CBCTs were truncated in a way that would significantly alter dose calculation (e.g. no samples were excluded based on patient characteristics, such as breast separation). CT simulation was performed after patients were immobilized on a commercially available prone breast board. A kV CBCT was taken before delivery of each fraction, as part of Halcyon v2.0 image-guided RT workflow. A total of 16 fx * 8 patients = 128 CBCT datasets were obtained. A synthetic daily CT was generated by deformably registering the planning simulation CT to each of the daily CBCT images using MIM Maestro® (MIM Software Inc., Cleveland, OH) version 6.6. CBCTs associated with boosts were excluded from daily evaluations.

The clinical treatment plans were generated using ECOMP technique (EZfluence™, Radformation, Inc, New York, NY) in Eclipse v15.6 (Varian Medical Systems, Palo Alto, CA), with dosimetric endpoints specified in Table 1. This plan was copied and recalculated on synthetic CT to obtain daily delivered dose distribution. BODY and Breast PTV_eval contours, as defined on Radiation Therapy Oncology Group (RTOG) trial 1005 [14] and census definitions [15] were created on each synthetic CT. The Breast PTV_eval contour was derived for each day of treatment, the process of which is clarified in the following subsections.

**Table 1:** Dose-volume objectives for Breast PTV Eval

Breast PTV Eval DVH objective	Evaluator (%)	Variation acceptable
D95%	≥ 95	≥ 90%
V90%	≥ 99	≥ 98%
V105%	≤ 10	≤ 15%/ ≤ 200 cc to Breast tissue [12]
Dmax	≤ 107	≤ 110%

### Dosimetric endpoints investigated

Most recent ASTRO guidelines on WBRT state that V105% should be minimized at all times, while some cite 200 cm^3^ as the recommended limit [12]. The tumor bed is to receive at least 95% of the prescription dose under ASTRO guidance. Similarly, RTOG1005 protocol stipulates (1) 95% of Breast PTV Eval shall receive at least 95% of prescribed dose (D95% > 95%, but D90% > 90% acceptable), and (2) maximum dose less than 115% of prescribed dose (Dmax < 115%, but 120% acceptable).

To investigate if delivered prone WBRT dose on Halcyon meets these guidelines on a per-fraction basis, three primary dose-volume metrics were measured at each fraction to assess PTV Eval coverage and hotspot: V90%, D95%, and V105%. Absolute values of V95% [cc], V100% [cc], and V105% [cc], and global maximum dose (Dmax) were obtained from the BODY contour. Predictive model for these dose-volume metrics were constructed, based on patient parameters including source-to-surface distance (SSD), couch shifts, residual breast position (defined below in Data Collection, Analysis, and Statistics section), and weight.

### Patient set-up and initial treatment planning

Eight (8) patients were simulated head-first-prone on Siemens Sensation CT scanner (Siemens Healthineers, Erlangen, Germany) for initial treatment planning. Patients were immobilized with QFix® Prone Breast (Avondale, PA) boards and a vac-lok bag placed underneath the patient and with their arms up. Imaging isocenter was placed midline of the body, at the midpoint (sup-inf) of the breast tissue and anteriorly to the sternum.

WBRT was planned in Varian Eclipse v15.6. Physicians contoured Breast CTV based on consensus definitions [15]. Breast CTV was expanded 5 mm (excluding heart and not crossing the midline) and cropped anteriorly from the skin by 5 mm and posteriorly in front of the rib to obtain Breast PTV Eval. Treatment isocenter was shifted anteriorly to cover Breast PTV Eval. ECOMP technique was carried out by experienced dosimetrists. ECOMP is a forward-planning intensity-modulated radiation therapy (IMRT) technique using parallel opposed beams, where the goal is to deliver as homogenous dose as possible to an irregular surface. For breast, this is done by planning a uniform dose at mid-separation. Skin flash of 2 cm was added beyond the patient contour, and then the edited fluence maps were converted to leaf sequences for Halcyon dynamic MLC. Machine energy was fixed at 6X-FFF (6-MV with flattening filter free), with prescription dose of 266 cGy/fraction for all patients. Anisotropic analytical algorithm (AAA) version 15.6.03 was used for volumetric dose calculation. For photon dose optimization and irregular surface compensator generation, photon optimizer version 15.6.03 was used.

Dose-volume constraints for Breast PTV Eval in a whole breast treatment used at our institution are listed on Table 1. D95% and V90% measure the extent of dose coverage to the breast, while V105% and Dmax are measures of dose hotspots. For this study, a < 200 cm^3^ objective is additionally applied for the entire patient volume (BODY contour) as another suggested measure of homogeneity by ASTRO consensus but was not a constraint used clinically during planning. Study of normal organs, such as the lungs, hearts, and contralateral breast were omitted from this study.

### Patient treatment workflow

For treatment on Halcyon v2.0 machines, patients were immobilized with identical QFix® Prone Breast boards and vac-loks used during simulation. Patients were scanned under Halcyon Breast protocol (125 kV, 491 projections at 10 mA and 10 ms, filtered back projection reconstruction) to obtain an on-board CBCT [10]. For 3D/3D matching, chest wall and ribs in the CBCT were aligned with those of the planning image, ensuring the breast in the CBCT is within the planning Breast PTV Eval contour. Online couch shift applied was saved in ARIA for all fractions. Patient body weight was acquired during the weekly on-treatment visit (OTV).

### CT deformation to daily CBCT and daily treatment planning

**Fig. 1:**
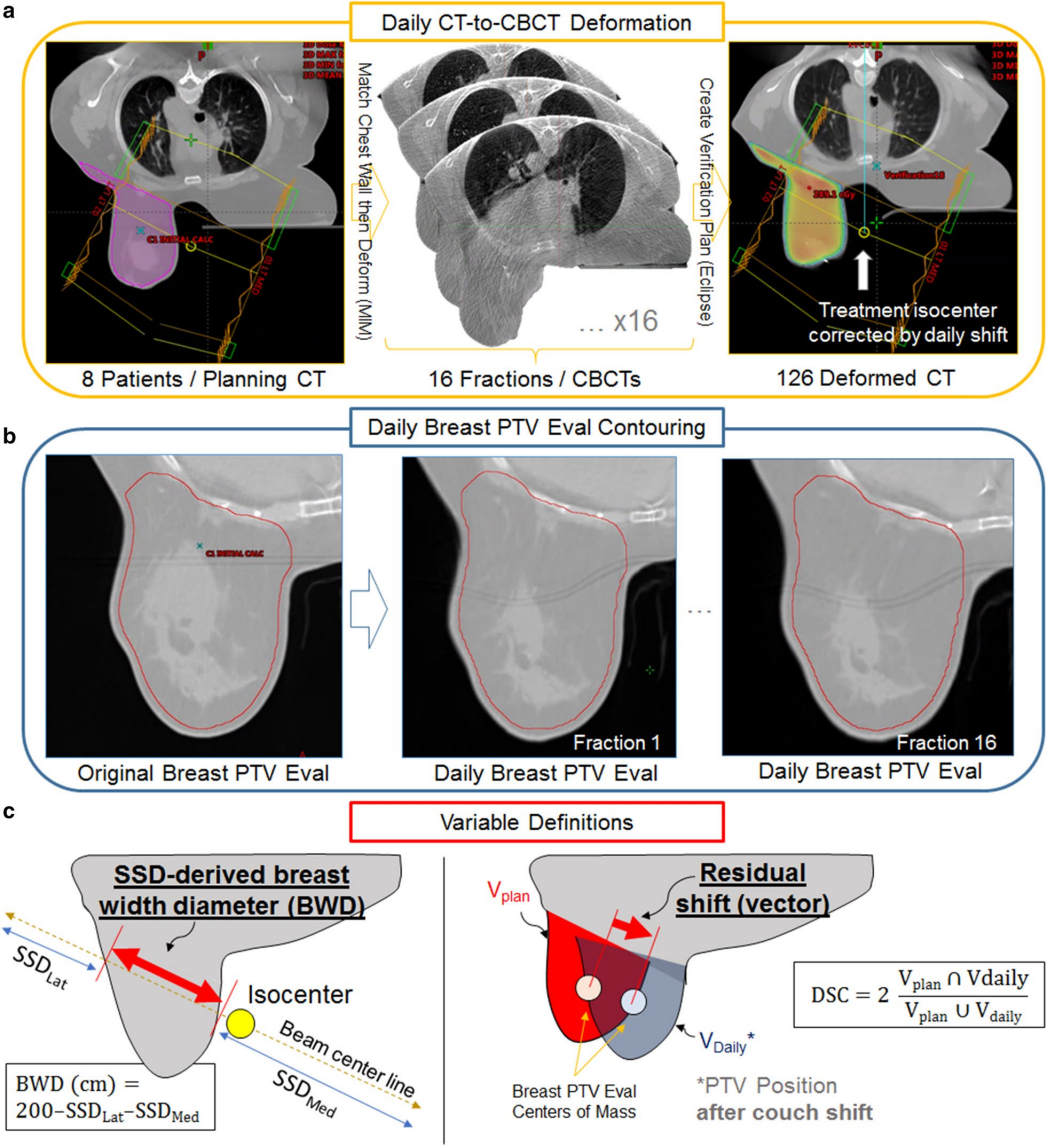
Illustration of daily synthetic CT generation and Breast PTV Eval contouring methods, as well as visual representation of variables used in predictive modeling. **a** Planning CT for 8 patients were deformed to each of 16 daily CBCTs taken by Halcyon v2.0. A total of 126 deformed CTs were generated. The daily shift from matching CBCT-CT was applied to copied plan isocenter to correct for daily couch shifts. **b** Generated Daily Breast PTV Eval mimicked the original contour as closely as possible, including a 5 mm subtraction from the skin surface. **c** Some predictive variables used in this paper are defined here. SSD-derived breast width diameter (BWD) is calculated from source-to-surface distances (SSD) of lateral and medial beams, giving an estimate of patient’s breast diameter as well as any set-up errors resulting in distorted SSDs. Residual shift vector was derived from comparing centers of mass of planned breast PTV eval volume to that of daily volume, adjusted for couch shift. Dice similarity coefficient (DSC) was calculated from this same setup using the intersection and union of PTV volumes

Figure 1a demonstrates the process to generate daily synthetic CTs using MIM Maestro v6.6. In the MIM console, planning CT was deformably registered to the CBCTs to generate synthetic daily CTs. Some synthetic CTs were discarded (2 in total) because of deformation failures stemming from limited CBCT field-of-view. A total of 126 synthetic CT images was extracted from MIM and imported back into Eclipse. Once imported, a copy of original RT plan was re-calculated on each of the synthetic daily CTs keeping all beam parameters intact, while taking into consideration the online CBCT-to-CT registration that was performed during daily treatment. Dose to synthetic daily CTs was calculated with the same AAA version as the original treatment plan.

Figure 1b demonstrates the contouring process on daily synthetic CTs for the treatment target, which is Breast PTV Eval contour. Not all PTV Eval contours were reviewed by physicians, but an effort was made to mimic the original contours from the planning CT in each daily synthetic CTs, by (1) starting with an actual copy of PTV Eval contour from planning CT rigidly registered to the synthetic CT chest wall, (2) modifying the contour to match to chest wall edge shape, and (3) applying a 5 mm subtraction from the breast tissue surface. The breast tissue surface was automatically determined by the Eclipse software in the contouring module for each of the synthetic CTs. Overall, this procedure ensures the PTV Eval structure generated on daily synthetic CT consistently represents the initial physician’s intent on breast tissue being irradiated for each daily fraction.

### Data collection, analysis, and statistics

We make a distinction between two categories of data: (1) planning data and (2) per-fraction/daily data. The former refers to the baseline parameters determined from planning CT and the planned dose. The latter refers to the treatment parameters determined from daily synthetic CTs and the *delivered* dose. The difference of daily data from planning data is denoted throughout this paper with an uppercase Greek delta (Δ). For example, ΔV105% = Daily V105% − Planned V105%.

Dosimetric endpoints of this study include the following. For Breast PTV Eval contour: V90% [%], D95% [%], and V105% [%]. For BODY contour: V95% [cc], V105% [cc], and global Dmax. Here, [%] means “percent of the contour volume” and [cc] means “absolute volume as centimeter cubed (cm^3^).” Dose-volume histogram (DVH) was calculated using DVH Estimation Algorithm version 15.6.03.

Patient positioning and volumetric data relevant to this study include the following. For general daily positioning data: lateral (LAT), vertical (VRT), and longitudinal (LNG) couch shifts [cm]; source-to-surface distances (SSD) [cm] for each of the two parallel opposed beams; breast width diameter (BWD) derived from SSD, as illustrated on Fig. 1; and post-image-guidance Breast PTV Eval shift [cm] (after accounting for daily couch shifts), referred to as “residual shifts” in this paper, also illustrated on Fig. 1. SSD-derived breast width diameter (BWD) gives a rough estimate of the breast diameter, but also captures some set-up errors of the day resulting in SSD distortions. For example, if both lateral and medial beam SSD increases for the day, BWD decreases; this can be interpreted as either breast diameter decrease or improper breast set-up resulting in breast compression. The residual shift is how much the Breast PTV center-of-mass shifts compared to during CT simulation, after taking into account the couch shifts. For volumetric data: Dice similarity coefficient (DSC) for breast deformations and Breast PTV Eval volume [cc]. DSC measures the extent of overlap between two contours, in our case daily Breast PTV Eval (constructed as shown on Fig. 1b) and planning Breast PTV Eval. DSC is equal to unity when a perfect overlap is achieved, and is calculated as such:

1 $${\text{DSC}} = \frac{{2{ }\left( {{\text{V}}_{{{\text{plan}}}} { } \cap {\text{ V}}_{{{\text{daily}}}} } \right)}}{{{\text{V}}_{{{\text{plan}}}} \cup {\text{V}}_{{{\text{daily}}}} }}$$

Other data collected in this study include machine data: collimator angles, gantry angles, and monitor units for each of the two parallel opposed beams. Patient weight data gathered during simulation and physician visits was spline-interpolated to estimate weight at every fraction.

All data collected were imported into MATLAB (Natick, MA) Version 2019a for data visualization and statistical analyses. Tables and histograms of predictor variables, dosimetric endpoints, and patient position information are presented with descriptive statistics. Non-parametric statistical tests (Wilcoxon signed-rank test) were used to compare daily delivered dose-volumes with dose-volume optimization criteria (Table 1) and with planned dose-volumes. Pearson correlation coefficients among all variables studied are presented, with emphasis on statistical significance of the observed correlation.

Finally, robust parallel slopes model (a type of analysis of covariance or ANCOVA) was constructed [16] to fit Δ dosimetric endpoints using the following predictors: couch shifts, PTV breast residual shifts, Δ BWD, 1-DSC, Δ PTV Eval Volume, and Δ body weight. In this model, each patient is assigned an intercept but the slopes with respect to predictors are assumed to be equal, hence the term “parallel slopes.” The general fitted formula was:

2 $${\text{Y}} = { }\upbeta _{{0{\text{i}}}} +\upbeta _{1} {\text{X}}_{1} + \cdots +\upbeta _{{\text{n}}} {\text{X}}_{{\text{n}}} +\upsigma$$

Here, ${\text{Y}}$ is the Δ dosimetric endpoints (e.g. ΔV105%), $\beta_{0i}$ is the intercept for *i*th patient, $\beta_{n}$ is the slope for *n*th predictor $X_{n}$, and $\sigma$ is a normally distributed residual term. The individualized intercept $\beta_{0i}$ for each patient attempts to compensate for patient-specific factors, such as the differences in optimized IMRT fields, machine parameters (gantry angles, monitor units), and other miscellaneous factors. A reasonable estimate of the slopes was made based on this model. Robustness of the model was improved with MATLAB’s default iterative robust least squares procedure using bi-square weights. This reduces effects of high-leverage data and outliers on the fit. Wald’s Test with the null hypothesis that $\beta_{n} = 0$ estimated statistical significance of each variable on the Δ dosimetric endpoints.

Significance was set at α = 0.05 for most statistical analyses except for multiple tests, for which Benjamini–Hochberg procedure was applied to limit false discovery rate [17].

## Results

### Descriptive statistics of positional and dosimetric data

Table 2 summarizes descriptive statistics of planned and daily patient positioning and contour volume data, as well as difference from prescribed (Δ). Positional data include couch shifts and PTV residual shifts in three orthogonal directions (latitude, vertical, and longitude) and their magnitudes (directional shifts summed in quadrature), source-to-surface distance (SSD) for both parallel opposed (lateral and medial) beams, and DSC comparing planned versus daily target volumes. Daily couch shifts were within ± 2.5 cm in each of the three principal directions, with a mean magnitude of shift of 1.19 ± 0.57 cm. Couch shifts of 0.68 ± 0.40 cm were previously reported in the literature for 21 patients treated on C-arm linac [18]. Breast positioning was consistent, with target center of mass shifting only by 0.26 ± 0.10 cm or 0.30 ± 0.15 cm (PTV Eval and V95% contour center of mass, respectively). The DSC roughly captures breast deformation taking into account the daily couch shifts. At DSC = 0.93 ± 0.02, the intersecting volume of daily and planned contours are 93% of their composite (union) volume and indicates a great set-up consistency. The ΔSSDs of lateral and medial beams were 0.08 ± 0.58 cm and 0.18 ± 0.42 cm from planned, respectively, with some ΔSSD > 1 cm. Patient weights varied from − 6.5% to + 4.7% compared to during CT simulation.

**Table 2:** Summary of daily volumetric and positional data

Variable	Value, median (range)	Mean ± Stdev
Contour volumes [cc]		
Breast PTV Vol	1095.20 (654.20–1343.70)	1040.21 ± 218.26
Breast PTV Eval Vol	899.90 (540.70–1149.80)	852.00 ± 196.96
Daily couch shift [cm]		
LAT	0.07 (− 2.26 to 2.28)	0.05 ± 0.92
LNG	− 0.13 (− 2.10 to 1.35)	− 0.12 ± 0.70
VRT	− 0.14 (− 2.20 to 1.93)	− 0.13 ± 0.63
MAG	1.08 (0.27–2.78)	1.19 ± 0.57
PTV Eval residual shift [cm]		
LAT	− 0.04 (− 0.35 to 0.32)	− 0.03 ± 0.12
LNG	0.07 (− 0.38 to 0.47)	0.05 ± 0.19
VRT	0.06 (− 0.32 to 0.43)	0.05 ± 0.13
MAG	0.25 (0.05–0.49)	0.26 ± 0.10
BODY V95% residual shift [cm]		
LAT	− 0.01 (− 0.60 to 0.46)	− 0.03 ± 0.22
LNG	− 0.04 (− 0.53 to 0.66)	− 0.04 ± 0.18
VRT	0.05 (− 0.58 to 0.43)	0.03 ± 0.16
MAG	0.27 (0.04–0.80)	0.30 ± 0.15
Daily SSD [cm]		
Lateral beam	85.45 (80.80–95.70)	86.24 ± 3.91
Medial beam	102.75 (94.70–104.80)	102.00 ± 2.82
Lateral beam deviation from planned	0.10 (− 2.10 to 1.30)	0.08 ± 0.58
Medial beam deviation from planned	0.10 (− 0.50 to 1.30)	0.18 ± 0.42
Target contour DSC		
Breast PTV Eval	0.93 (0.88–0.97)	0.93 ± 0.02
BODY V95%	0.92 (0.88–0.96)	0.92 ± 0.02
Patient weight [%]		
Deviation from planned/simulation	− 0.48 (− 6.50 to 4.71)	− 0.64 ± 2.69

cc, centimeter cubed (cm^3^); PTV Eval, planning treatment volume for evaluation; LAT, lateral; VRT, vertical; LNG, longitudinal; MAG, magnitude; SSD, source-to-surface distance; DSC, dice similarity coefficient; Stdev, standard deviation

**Fig. 2:**
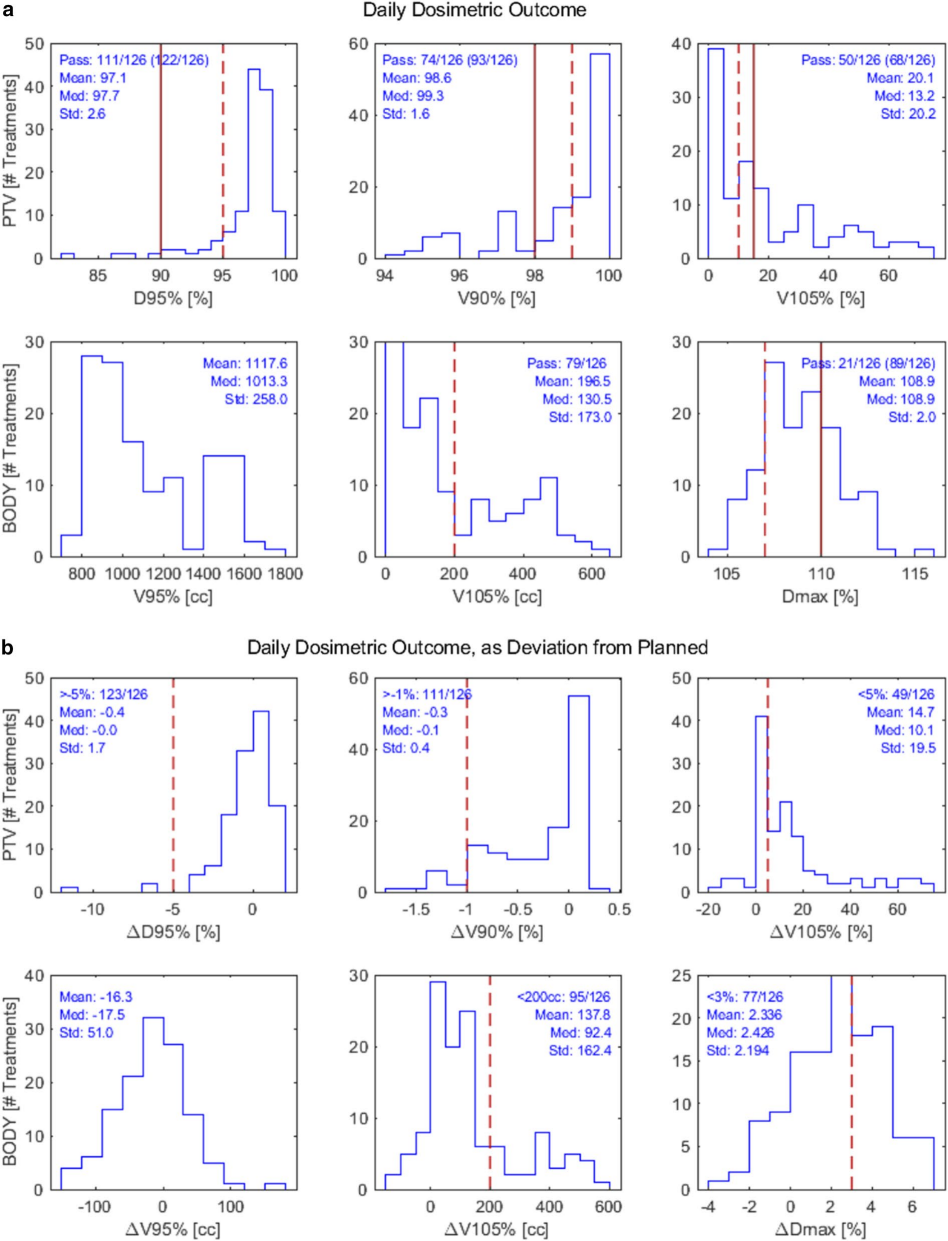
Histograms of daily dosimetric outcome based on Breast PTV Eval and BODY contours. Red vertical lines represent dose-volume constraints as defined on Table 1 (dashed: treatment objectives, solid: variation acceptable). Abbreviations: Med = median; Std = Standard deviation. **a** Top row: dosimetric endpoints for the Breast PTV Eval contour. Bottom row: for BODY contour. Number of treatments that meet the dose-volume objectives laid out on Table 1 is represented as a fraction out of 126, in the following format ‘N/126 (M/126)’ where N = # meeting objectives and M = # within the variation acceptable. **b** Corresponding histograms of daily dosimetric outcome, represented as difference from what was originally planned for the treatment courses. Number of treatments that do not deviate significantly from planned (N) are represented in format ‘N/126’, where “significant deviation” means “minimum deviation that would cause a plan meeting all dosimetric endpoints to extend beyond variation acceptable

Table 3 tabulates the six delivered dosimetric parameters for homogeneity (Breast PTV Eval V90%, D95%, V105%, BODY V95%, V105%, and global max dose) planned, daily delivered, and difference from prescribed/planned (Δ). Wilcoxon *p* values indicate the probability that the median value meets the dose-volume objectives (set out on Table 1) for daily measurements or the probability that the median Δ is non-zero. While target coverage was stable, delivered dose had hotspots failing to meet objectives and varying from prescribed dose frequently. The median of daily PTV V105% [%] failed to meet the < 10% objective, at 13.15%. Median global max dose [%] was found to be > 107% at 108.85%, but BODY V105% [cc] was found to be < 200 cm^3^. Median values of all quantities were within the variation acceptable (see Table 1), but some were not within the treatment objective. Figure 2 shows histograms of daily delivered dosimetric outcomes and difference from prescribed (Δ). The number of treatments that meet the prescribed dosimetric endpoints (i.e. “pass”) or has a small Δ (e.g. “< 10%”) are represented as a fraction out of all treatments (126) examined. The histograms corroborate that target coverage metrics were stable, but dose hotspot metrics were not. Dose objective D95% > 95% was met 111 days out of 126 daily CTs examined, while V90% > 99% was less frequently met at 74/126 (but 95/126 within variation acceptable, V90% > 95%). For difference from prescribed, ΔD95% was > -5% for 123/126 days and ΔV90% was > -1% for 111/126 days. BODY V90% deviated only slightly from planned, with a standard deviation of ± 51.0 cc with a bias of − 16.3 cc. In contrast, PTV V105% < 10% was met only 50/126 days (68/126 within variation acceptable). Dmax was the most often not met, with only 21/126 days meeting the criteria of < 107% of the prescribed dose.

**Table 3:** Summary of planned dosimetric parameters and day-to-day dosimetric results

Variable	Value, median (range)	Wilcoxon *P* value
Planned dosimetric quantities		
Breast PTV Eval		
D95% [%]	97.73 (93.63–98.64)	–
V90% [%]	99.50 (95.50–100.00)	–
V105% [%]	0.10 (0.00–41.10)	–
BODY		
V90% [cc]	1174.64 (863.13–1698.72)	–
V105% [cc]	3.67 (0.00–390.63)	–
Global max dose [%]	105.59 (104.94–106.92)	–
Daily dosimetric quantities		
Breast PTV Eval		
D95% [%]	97.74 (82.56–99.51)^a^	N.S
V90% [%]	99.30 (94.20–100.00)^a^	N.S
V105% [%]	13.15 (0.00–72.50)^b^	1.5 × 10^−4^
BODY		
V90% [cc]	1107.75 (831.38–1905.60)	–
V105% [cc]	130.45 (0.00–640.39)^a,c^	N.S
Global max dose [%]	108.85 (104.92–115.08)^b^	4.4 × 10^−16^
Difference from prescribed (Δ)		
Breast PTV Eval		
ΔD95% [%]	− 0.04 (− 11.07 to 1.78)	N.S
ΔV90% [%]	− 0.10 (− 1.70 to 0.20)*	6.1 × 10^−11^
ΔV105% [%]	+ 10.10 (− 15.40 to 72.50)*	6.3 × 10^−16^
BODY		
ΔV90% [cc]	− 18.07 (− 155.14 to 206.87)*	3.4 × 10^−3^
ΔV105% [cc]	+ 92.40 (− 120.24 to 596.96)*	9.4 × 10^−18^
ΔGlobal max dose [%]	+ 2.43 (− 3.02 to 6.59)*	6.7 × 10^−17^

cc, centimeter cubed (cm^3^); PTV Eval, planning treatment volume for evaluation; N.S., not significant

^*^Significantly different from planned (two-tailed Wilcoxon)

^a^Median value meets objective (one-tailed Wilcoxon)

^b^Median significantly lower/higher than objective (one-tailed Wilcoxon)

^c^BODY V105% does not have an objective, but ASTRO recommends < 200 cm^3^ to breast

### Dosimetric performance per patient

**Fig. 3:**
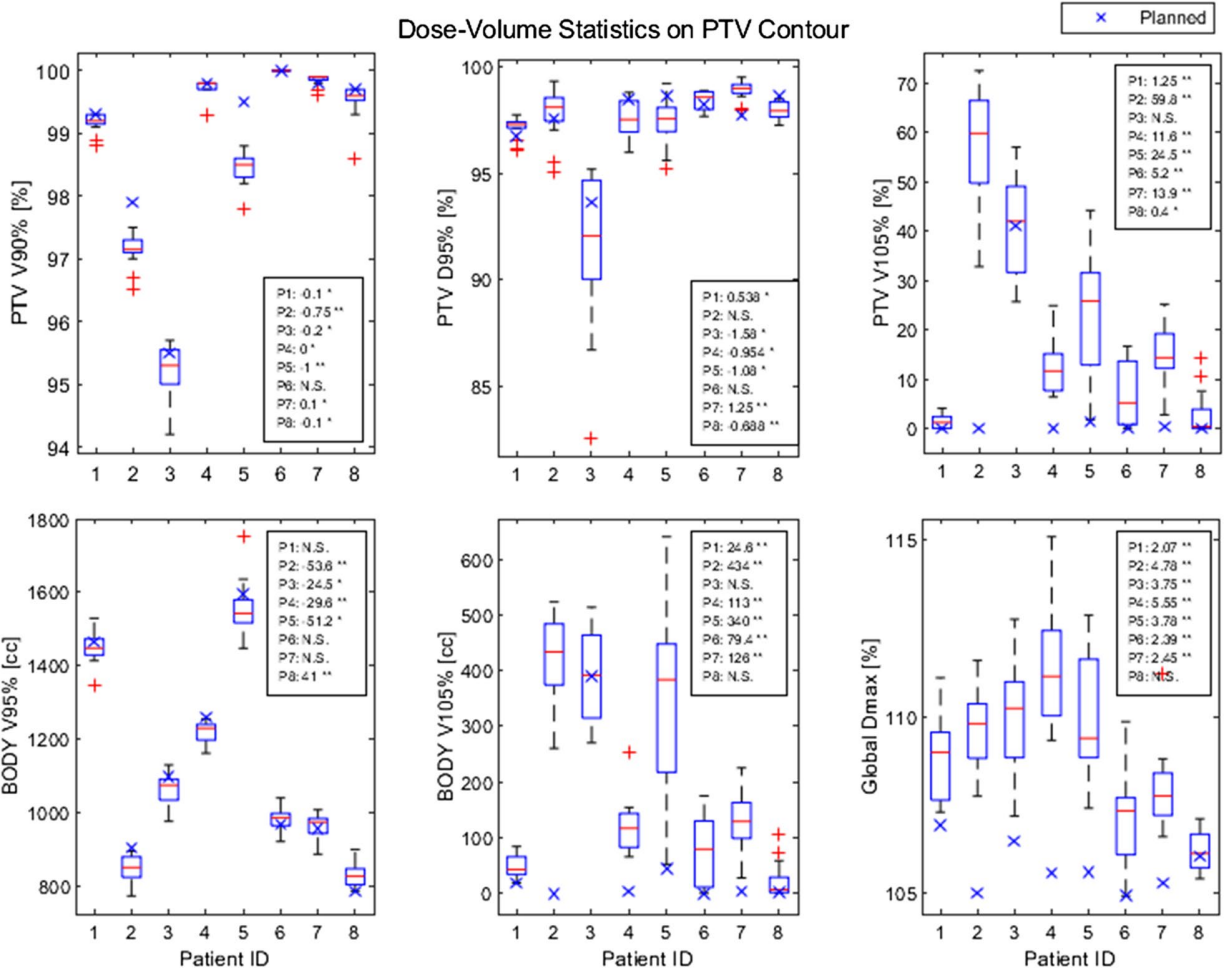
Boxplot of dose-volume quantities over the course of 16 fractions. Blue X represents planned quantities for each patient. Red + represents outlier fractions over the course of 16 fractions. Inset box: Median difference between planned quantities and daily quantities, with associated *p* value (Wilcoxon signed-rank test) for each patient P1–P8. Statistical abbreviations: N.S. = not significant (*p* > 0.05); * = significant (0.001 < *p* < 0.05); ** = very significant (*p* < 0.001)

Figure 3 presents PTV V90%, D95%, V105%, BODY V95%, V105%, and global Dmax over the course of 16 fractions as a boxplot. Most metrics deviated from their planned values (indicated as a blue X) with statistical significance indicated within the parenthesis. Measures of coverage (V90%, V95%, and D95%) sometimes decreased during treatment versus planned (Δ < 0). Measures of dose hotspots (V105% and Dmax) generally increased during treatment versus planned (Δ > 0). Some patients, such as patients 2 and 5, experienced both decrease in coverage and increase in dose hotspots.

### Correlation of variables and multiple linear regression

**Fig. 4:**
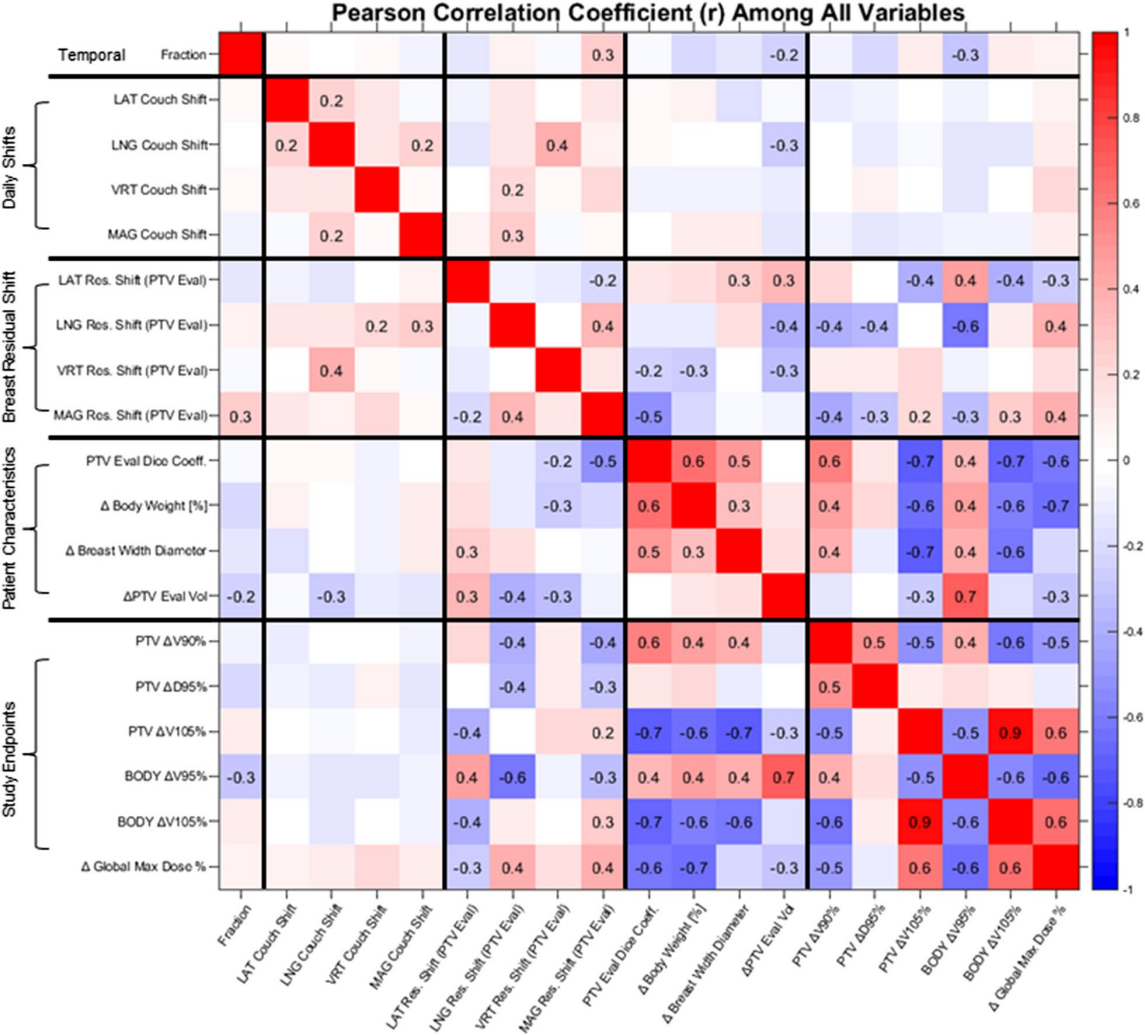
Pearson correlation coefficients among all data gathered. Negative correlations are represented by blue color. Positive correlations are represented by red color. Pearson correlation coefficient (r) is shown only when statistically significant (significance set to α = 0.0177 via Benjamini–Hochberg procedure for multiple testing)

Figure 4 depicts Pearson correlation matrix among 21 variables studied in this paper, with only statistically significant Pearson coefficients (*p* > 0.0177) displayed after multiple testing corrections. Negative correlations are colored blue and positive correlations are colored red. The first variable (column or row) represent change with respect to time (fraction number). The next 8 variables represent daily couch shifts, followed by residual shift of the breast center-of-mass. The next 4 variables represent daily patient geometry: dice coefficients (deformation) of Breast PTV Eval contours, SSD-derived BWD, weight, and Breast PTV Eval volume. Remaining 6 parameters are the study endpoints: PTV and BODY dose-volume data. The uppercase Greek delta (Δ) denotes change with respect to planned or simulated (for example, Δ Body Weight [%] means change in patient body weight compared to during simulation).

The first column (or row) shows trends of each variable with respect to time. The magnitude of Breast PTV Eval residual shift increased with time (r = + 0.3), while volumes of PTV Eval and V95% decreased with each fraction (r = − 0.2 and − 0.3, respectively). Breast volume decreased on average − 2.21 and − 3.05 cc per fraction for Breast PTV Eval and Body V95% respectively (*p* = 0.026 and 0.0018, Robust linear regression). Daily couch shifts, where LAT is positive towards patient right (in prone position), LNG is positive towards patient head, and VRT is positive towards patient posterior (again, in prone position), correlated little with dosimetric endpoints. Some couch shifts correlated with residual shifts. Interestingly, LNG couch shift correlated with PTV Eval Volume (r = − 0.3). Breast deformation (DSC), body weight, and BWD correlated heavily with each other, as well as with dosimetric endpoints. Breast PTV Eval DSC correlated strongly with body weight (r = + 0.6), suggesting that breast deformation increases with decreasing body weight. Body weight positively correlated with BWD, albeit weakly (r = + 0.3).

**Fig. 5:**
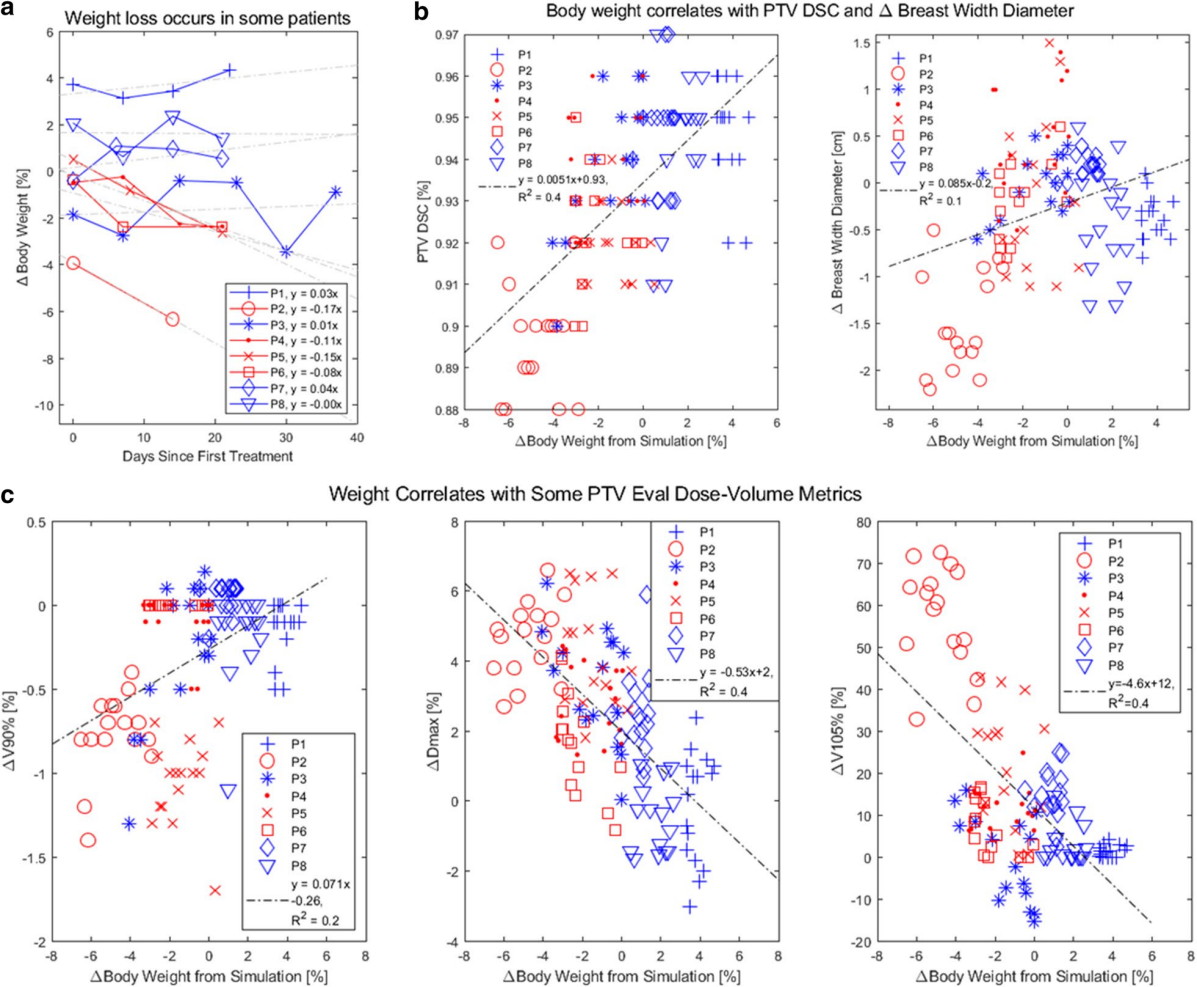
Body weight correlates with DSC, BWD, and PTV dose homogeneity and hotspot metrics. Data from patients who consistently lost weight over the course of the treatment are denoted in red (P2, P4, P5, and P6), while others are denoted blue. Linear fits (dotted lines) with equations and regression coefficient are also shown. **a** Some patients were susceptible to weight loss, with one patient (P2) losing 4% body weight from simulation even before treatment began. **b** DSC and BWD correlated to body weight. **c** Breast PTV Eval ΔV90%, ΔDmax, and ΔV105% (Δ = change from planned) correlates well with Δ body weight. Weight loss correlated to decreased PTV V90%, increased Dmax, and increased V105%, highlighting its crucial role in target coverage and reduction of hotspot

Correlation of body weight with DSC, BWD, and dosimetric endpoints are plotted in Fig. 5. While some patients lost weight, some maintained weight throughout the course of treatment. Weight loss decreased DSC (or increased breast deformation) and moderately decreased BWD. The change in body weight from simulation (Δ) correlated positively with metrics of dose coverage like PTV ΔV90% (r = + 0.4, slope of 0.071% per weight %) and negatively with metrics of hotspot like ΔV105% (r = − 0.6, slope of − 4.6% per weight %).

The results of comprehensive robust multiple linear regression fit are presented on Additional files 1 and 2. Additional file 1 tabulates the slopes ($\beta_{n}$) estimated for each variable. Additional file 2 tabulates the estimated intercepts ($\beta_{0i}$) for *i*th patient and the overall adjusted R^2^ for the model. Each model predicts Δ dose-volume metric. ΔD95% was excluded due to lack of daily plans providing large variations in D95% which resulted in a poor fit (low adjusted R^2^ = 0.4).

**Fig. 6:**
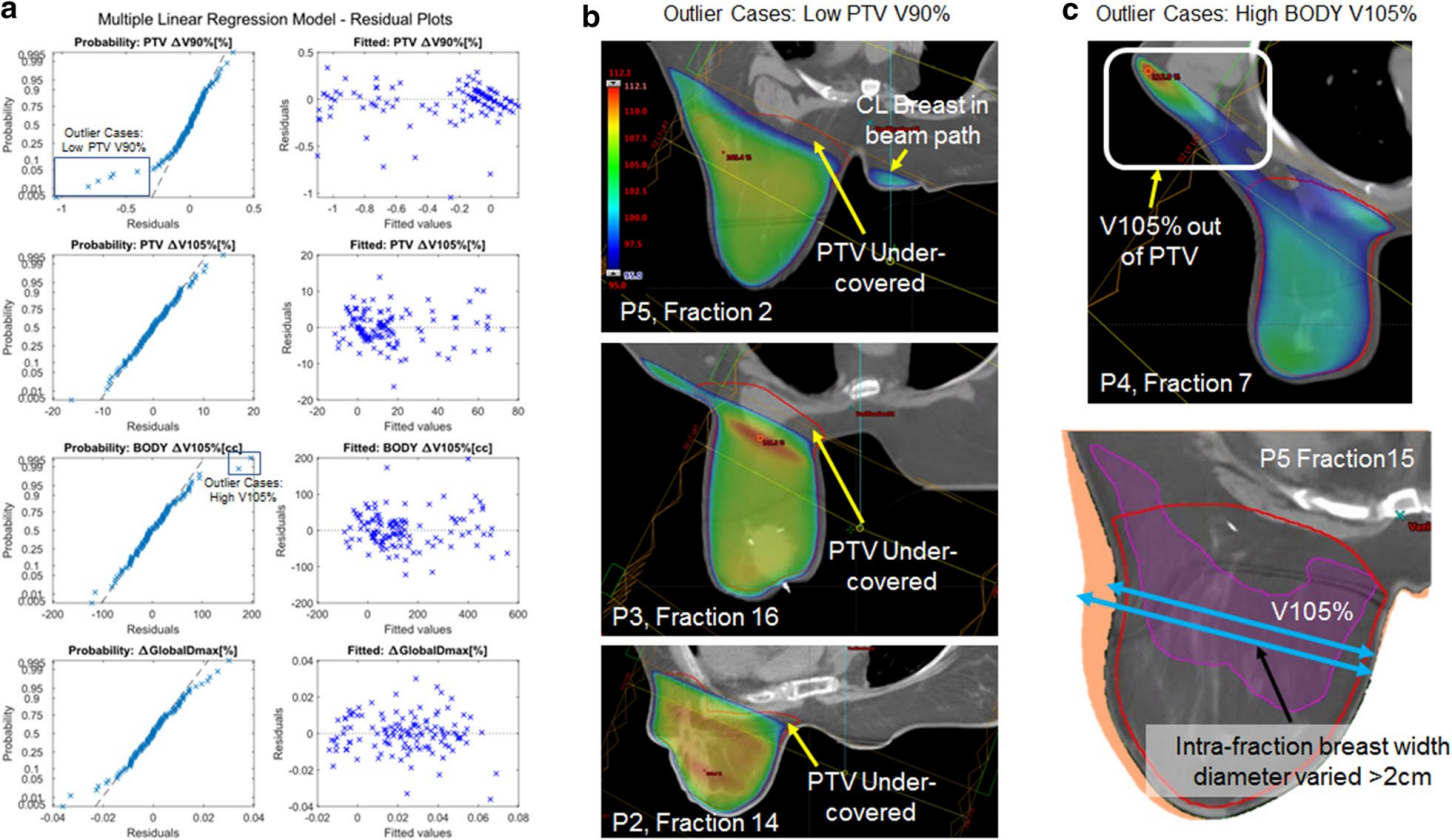
Robust parallel slopes model residual (σ) plots and examination of model outliers. **a** Residual normal probability plot and residual versus fitted values plot. Residuals did not necessarily follow a normal distribution, for example for PTV ΔV90% [%] and BODY ΔV105% [cc]. Residuals were homoscedastic. **b** Representative outlier cases where PTV ΔV95% [%] was exceptionally low. Dose map ranges from 95% of prescription dose (blue) to respective maximum dose (red). PTV under-coverage occurred mostly at the chest wall. The beams were also very close to the contralateral (CL) breast and in one of the cases (patient 5, fraction 2) were irradiated outright above 95% of the prescription dose. **c** Representative outlier cases where BODY ΔV105% was exceptionally high. In the top case (patient 4, fraction 7), an area outside of the PTV contour was discovered that unexpectedly increased the BODY ΔV105%. In the bottom case (patient 5, fraction 15), the breast was highly deformed compared to the previous fraction (orange)

Figure 6 summarizes the behavior of residuals and outliers of the robust parallel slopes model described on Additional files 1 and 2. Residuals were mostly normally distributed, but some outliers had residuals deviating more than expected indicating non-linear effects. Outliers for PTV ΔV90% delivered lower-than-expected dose due to simply missing the Breast PTV Eval near the chest wall. Many of these outliers had contralateral breast in the line of irradiation, perhaps indicating there were clinical decisions to avoid irradiating the contralateral breast. Outliers for BODY ΔV105% either had irradiated volume outside of Breast PTV Eval artificially increasing ΔV105% or had extreme variations (> 2 cm) in intra-fraction BWD.

## Discussion

Overall, the metrics of dose coverage such as PTV D95% [%], PTV V90% [%], and BODY V95% [cc] are both typically within the variation acceptable and robust on a day-to-day basis for prone-positioned breast cancer patients treated with tangential radiotherapy on Halcyon. Evidence supporting both claims can be most clearly seen in Fig. 2. On the other hand, metrics of radiation hotspot such as V105% and global hotspot (Dmax) met the prescribed objectives less often, as seen on Fig. 3. In some patients (2 and 5) the BODY V105% was consistently > 200 cc on a daily basis even when the planned dose met this constraint, while the other 6 patients in this study consistently met the criteria.

Patient body characteristics such as weight, SSD-derived BWD, and breast volume may explain elevated V105% and Dmax in some of these patients. For example, on Fig. 5, intra-patient ΔDmax increased by 0.53% and PTV ΔV105% increased by 4.6% for every 1% weight loss from CT simulation (reminder that Δ signifies difference from prescribed). Patient 2 lost ~ 4% of her weight since CT simulation even before treatment began and 2% more during the course of the treatment. It is possible that dose delivered to patient 2 failed to meet dose constraints due to the significant weight loss.

Weight loss likely correlates with ΔV105% and ΔDmax because it decreases breast deformation (DSC) and BWD. In our comprehensive parallel slopes model (Additional file 1), reduced DSC and BWD resulted in increased ΔV105% and ΔDmax. Since weight loss decreases BWD and DSC (Figs. 4, 5b), weight loss should increase ΔV105% and ΔDmax according to the parallel slopes model. Interestingly, weight was not a significant predictor of ΔV105% and ΔDmax when both BWD and DSC was present. It is possible that including these two variables in the model effectively cancel out the influence of weight loss on ΔV105% and ΔDmax.

DSC and BWD reduction from weight loss were not the sole cause of ΔV105% and ΔDmax increase. In our model, PTV eval volume also significantly influenced intra-patient variability in ΔV105% and ΔDmax. Surprisingly, weight did not correlate with PTV eval volume (Fig. 4). This is interesting since breast volume is expected to decrease with weight loss. It is possible that post-lumpectomy seroma resolution is decoupling breast volume from weight loss. Indeed, PTV eval volume as a whole was also observed to decrease with time (r = − 0.2) in Fig. 4, suggesting gradual seroma resolution over the treatment course.

In our model for dose coverage metric PTV ΔV90%, BWD, weight, and PTV Eval volume influences dose coverage along with some positional shifts (namely: vertical couch shift, longitudinal residual shift, and magnitude of residual shift). It is unclear why these specific couch and residual shifts decrease PTV ΔV90%. It is possible that negative vertical couch movements (i.e. couch moves down) increase PTV ΔV90% since that encourages more of the PTV Eval Breast to be within the field. It is also possible that residual shifts decrease PTV ΔV90%, because any post-couch-shift residual movement of the breast will knock the breast out of the confines of the parallel-opposed field. Nevertheless, the slopes of these predictors with respect to PTV ΔV90% are quite small compared to PTV ΔV105%, again corroborating that ΔV90% is robust.

A Halcyon-specific guideline to reduce hotspots in can be established based on the conclusions from parallel slopes model to ensure dose hotspot is limited and dose coverage is maximized. First, patient’s weight and breast volume should be monitored closely for any extreme changes. Second, if patient weight loss or breast volume changes is over a certain threshold, BWD and breast volume should be re-examined during daily CBCT. A re-plan should be considered if BWD or breast volume changed significantly and would result in excessive hotspots or a significant loss in dose coverage.

**Fig. 7:**
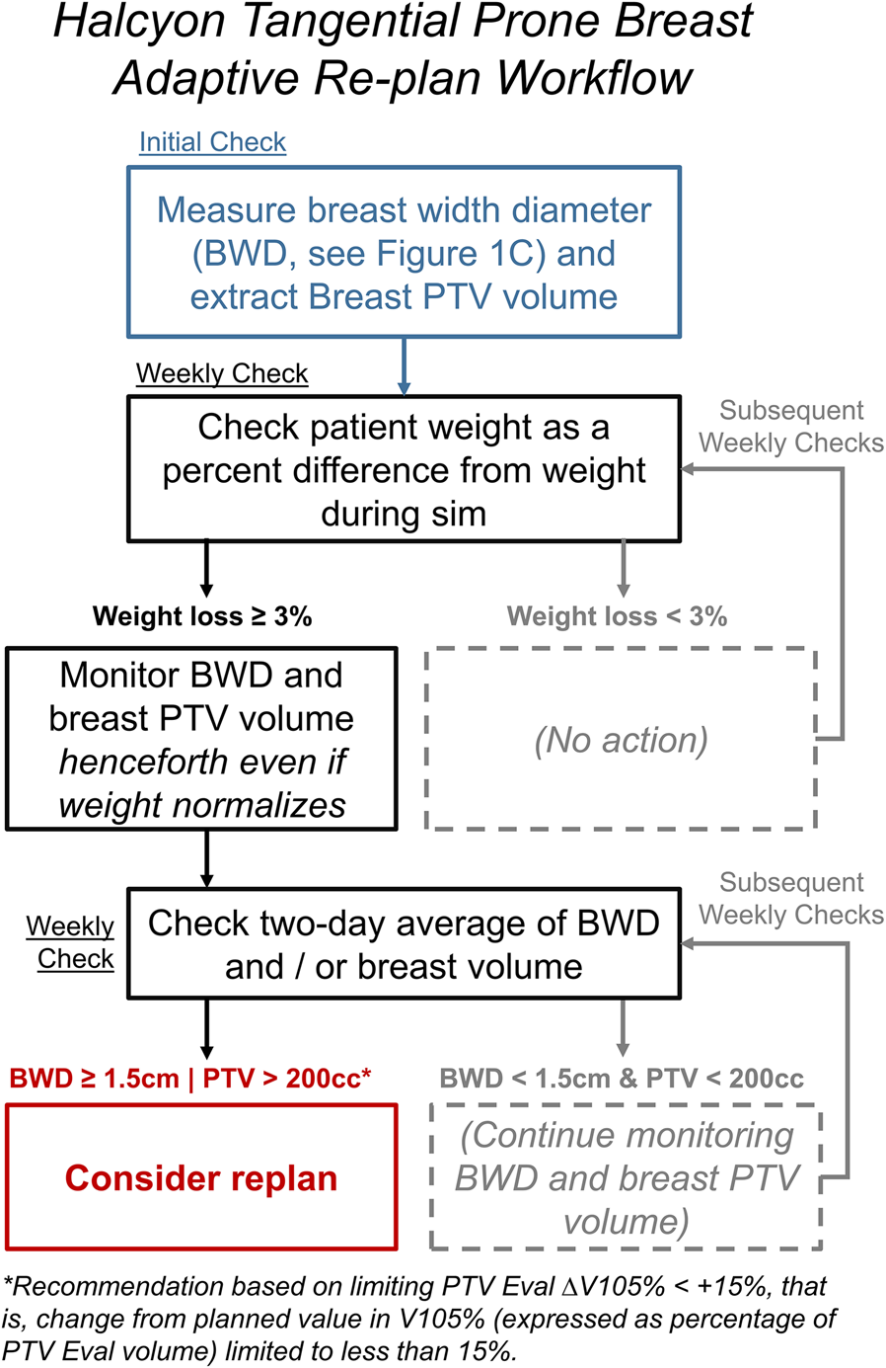
Flowchart for preliminary recommendations for prone breast adaptive re-planning on Halcyon. Depending on capabilities and workflow of one’s institution, one may skip monitoring weight and just monitor breast width diameter (BWD) or breast volume. Suggested timing (initial or weekly) to check weight or BWD/breast volume is also indicated. Dotted boxes indicate no action taken

The following are preliminary recommendations (pictorially described on Fig. 7) based on the estimated parameters from the model established on Additional files 1 and 2 and on the dose-volume objectives laid out on Table 1 during the course of the treatment:

- During CT simulation and planning, measure breast width diameter and extract breast volume from Breast PTV (not Breast PTV Eval).
- If a patient lost weight by > 3% compared to during simulation, re-measure breast width diameter and breast PTV volume of the patient in prone position henceforth, even if the patient’s weight normalizes.
- If a two-day average of either patient’s breast width diameter or breast volume deviates more than 1.5 cm or 200 cc compared to during CT simulation, respectively, consider a re-plan for the subsequent fractions.

The goal of the recommendations above is to limit per-fraction PTV ΔV105% (change from planned) to < + 15%, without significantly affecting clinical workflow and treatment time. Note that, because BWD is derived from SSD, it is subject to set-up errors. The two-day average helps smooth out the daily variations due to set-up errors. Weight is a metric that is easy to measure during weekly on-treatment visits and can be obtained more frequently easily. Since 1% weight loss results in 4.6% increase in PTV ΔV105%, 3.3% weight loss corresponds to PTV ΔV105% of + 15% (rounded down to 3%). Additional file 1shows that PTV V105% increases by 10.1% (BODY V105% by 83.2 cc) for every 1 cm decrease in BWD, all other variables being equal. PTV V105% increases by + 8.79% (BODY V105% by + 86.2 cc) for every 100 cc decrease in breast volume. That meant a 200 cc increase in Breast PTV (not breast PTV eval) leads to about + 15% increase in PTV V105%, taking into account the PTV Eval to PTV volume ratio of ~ 1.21. Note that, if weight cannot be measured regularly, one can skip measuring the weight and monitor the two-day averages of breast width diameter (as defined in Fig. 2) and volume daily.

There are several limitations with the model despite the high adjusted R^2^ reported (0.94 for V105% and 0.84 for V90%). The $\beta_{0i}$ intercept estimates (Additional file 2) show inter-patient biases are quite significant for V105% and Dmax that cannot be fully explained by the $\beta_{n}$ slope components of the regression. It is expected that all slopes should be zero if there were no patient-to-patient biases that predisposes one patient to receive higher or lower dose. There may be second order effects, interaction effects (i.e. variable slopes per patient rather than a single slope), or simply effects from personalized optimized IMRT fields that explain the non-zero intercepts. Another explanation could be that dose-volume metrics like V90% and V105% are inherently non-linear. Whatever the case may be, slopes on Additional file 1 should only be used to estimate intra-patient variations (i.e. fraction to fraction), never inter-patient variations.

Another limitation is that the model is statistically low-powered (n = 126, but only 8 patients) and fails to explain a few outlier or non-linear behaviors. In addition, despite the use of robust methods, our results may have been affected by patient 2, who experienced disproportionately severe weight loss than other patients in the trial. There were outlier behaviors the model did not predict as well, such as significant under-dosing (i.e. low PTV V90%) close to the chest wall for some fractions. For the two cases where BODY V105% [cc] was exceptionally high, there was an unexpected high-dose area near the arm outside of PTV for one patient (P4, fraction 7) and a significantly decreased breast size for another (P5, fraction 15).

## Conclusion

We examined and modeled the daily behavior of dose homogeneity for tangential field prone-positioned WBRT, using daily Halcyon v2.0 kV CBCT. The goal was to assess day-to-day robustness of electronic tissue compensation (ECOMP) technique with 6X-FFF on Halcyon. While metrics of dose coverage such as V90% was often met, metrics of dose hotspot such as V105% and Dmax routinely exceeded specified dose constraints on a per-fraction basis for select patients. Data suggests inter-patient differences in dose hotspot could come from patient body characteristics, such as weight, breast volume, and SSD-derived breast width diameter (BWD). In the comprehensive model, three factors explained most intra-patient variations in V105% and Dmax: breast volume, BWD, and breast deformation compared to planned. For some patients, severe weight loss affected breast width diameter and breast deformation, resulting in higher V105%’s than planned. Some patients also experienced breast volume losses independently of weight, possibly due to seroma resolution, that significantly increased V105% and moderately increased Dmax. Data suggests that some patients vulnerable to extreme weight loss or breast volume changes from seroma resolution may benefit from re-planning.

## Supplementary information

**Additional file 1:** Parallel slopes model: effect of clinically relevant predictors on dose-volume metrics deviation from planned, effect size per unit.

**Additional file 2:** Parallel slopes model: effect of clinically relevant predictors on dose-volume metrics deviation from planned, estimation of per-patient intercepts and overall regression coefficient.

## Data Availability

The datasets used during the current study are available from the corresponding author on reasonable request.

## Abbreviations

WBRT: Whole breast radiation therapy
6X-FFF: 6MV flattening filter free
ECOMP: Electronic compensation
PTV: Planning treatment volume
BWD: Breast width diameter
DSC: Dice similarity coefficient
BCT: Breast conservation therapy
EPID: Electronic portal image devices
FiF: Field-in-field
MLC: Multi-leaf collimator
RTOG: Radiation Therapy Oncology Group
cc: Centimeter cubed (cm^3^)
SSD: Source-to-surface distance
CTV: Clinical target volume
AAA: Anisotropic analytical algorithm
DVH: Dose-volume histogram
LAT: Lateral
VRT: Vertical
LNG: Longitudinal
MAG: Magnitude
N.S.: Not significant
ANCOVA: Analysis of covariance
